# A nomogram integrating the CHA_2_DS_2_-VASc score for predicting atrial fibrillation recurrence following catheter ablation

**DOI:** 10.1186/s12872-025-05071-2

**Published:** 2025-10-01

**Authors:** Lin-Qian Jiang, Yu-Hong Zhong, Xue-Hai Chen, Zhe Xu, Ke-Zeng Gong, Fei-Long Zhang

**Affiliations:** 1 Department of Cardiology, Fujian Clinical Medical Research Center for Heart and Macrovascular Disease, Fujian Medical University Union Hospital, Fujian Heart Medical Center, Fujian Institute of Coronary Heart Disease, 29 Xinquan Road, Gulou District, Fuzhou, 350001 China; 2 Department of Intensive Care Unit, Fujian Provincial Hospital South Branch, The Shengli Clinical Medical College of Fujian Medical University, Fuzhou University Affiliated Provincial Hospital, Fuzhou, 350028 China; 3 Department of Cardiology, Huian County Hospital, Quanzhou, 362100 China

**Keywords:** Atrial fibrillation, CHA_2_DS_2_-VASc score, Catheter ablation, Nomogram, Recurrence

## Abstract

**Objective:**

This study aimed to explore the predictive efficacy of a nomogram based on the congestive heart failure, hypertension, age, diabetes mellitus, prior stroke, transient ischemic attack or thromboembolism, vascular disease, age, and sex category (CHA_2_DS_2_-VASc) score in predicting atrial fibrillation (AF) recurrence following first-time catheter ablation in patients with symptomatic AF.

**Methods:**

Clinical data of 398 patients were collected and analyzed. Patients were divided into a recurrence group (*n* = 81) and a non-recurrence group (*n* = 317). Key predictive factors were identified through univariate and multivariate analyses, and a nomogram was subsequently constructed using the R programming language.

**Results:**

The duration of AF, recurrence during the blanking period, neutrophil granulocyte count, neutrophil-to-lymphocyte ratio (NLR), red blood cell distribution width, and left atrial diameter (LAD) were identified as independent risk factors for AF recurrence (*p* < 0.05). A predictive model incorporating the CHA_2_DS_2_-VASc score, AF duration, NLR, and LAD was constructed. Among these variables, NLR exhibited the highest predictive value for postoperative recurrence of AF, followed by LAD, AF duration, and the CHA_2_DS_2_-VASc score. The concordance index (C-index) of the nomogram was 0.707 (95% CI: 0.566–0.847), which was significantly higher than that of the CHA_2_DS_2_-VASc score (C-index: 0.499; 95% CI: 0.359–0.640). The prediction model that was developed demonstrated clinical utility for assessing the risk of late recurrence across different AF subtypes and ablation techniques (AUC > 0.5).

**Conclusion:**

A nomogram incorporating the CHA_2_DS_2_-VASc score was developed to predict the recurrence of AF following ablation. It demonstrated promise in predicting the probability of recurrence 12 months post-ablation. However, further validation is required to confirm its reliability and generalizability.

## Introduction

Atrial fibrillation (AF) is one of the most common arrhythmias in clinical practice, with its incidence steadily increasing each year. The associated complications, including stroke, thromboembolism, and heart failure, pose serious threats to both health and quality of life [1]. In 2024, the European Society of Cardiology (ESC) introduced a comprehensive approach for the management of AF [2]. Rhythm control strategies mainly include pharmacological cardioversion, electrical cardioversion, and catheter ablation. Catheter ablation is currently the most effective means of restoring sinus rhythm in AF and is the recommended treatment for AF in China as well as per international guidelines. However, there is still a certain risk of recurrence that persists following catheter ablation. According to the Sustained Treatment of Paroxysmal Atrial Fibrillation (STOP-AF) study, early AF recurrence was observed in approximately 51.5% of patients undergoing cryoballoon ablation, while late AF recurrence occurred in 25.1% [3]. 

As medical knowledge continues to advance, numerous factors have been identified as valuable predictors for the recurrence of AF following catheter ablation. The congestive heart failure, hypertension, age, diabetes mellitus, prior stroke, transient ischemic attack (TIA) or thromboembolism, vascular disease, age, and sex category (CHA_2_DS_2_-VASc) score was originally developed to assess stroke and thromboembolic risk in patients with AF [4]. The latest guidelines no longer consider the sex category as a risk factor in the CHA₂DS₂-VASc score. Among these components, hypertension and diabetes mellitus have been reported to be associated with AF recurrence. Therefore, the CHA_2_DS_2_-VASc score has been considered a potential prognostic tool for AF recurrence following catheter ablation [5]. However, in recent studies where the predictive value of various scoring systems was analyzed, it has been suggested that the CHA_2_DS_2_-VASc score lacks predictive utility for AF recurrence following radiofrequency ablation. At present, several studies have focused on identifying factors influencing atrial remodeling and the pathophysiological mechanism underlying AF to improve the prediction of AF recurrence [6, 7]. 

The aim of the present study is to identify relevant predictors of AF recurrence through univariate and multivariate analyses of various risk factors, develop a nomogram model incorporating the CHA_2_DS_2_-VASc score to predict AF recurrence following ablation, and evaluate the predictive accuracy and clinical applicability of this model to facilitate individualized risk assessment and improve post-ablation management.

## Materials and methods

### Study participants

In this study, patients with AF who visited the Department of Cardiology in our hospital between June 2019 and June 2021 were included. A total of 398 patients with symptomatic AF who underwent their first transcatheter ablation and were regularly followed up were enrolled based on the inclusion and exclusion criteria. As per the type of AF, there were 260 patients with paroxysmal AF and 138 patients with persistent AF. Prior to treatment, all patients signed an informed consent form, and the study was approved by the local ethics committee.

### Inclusion criteria

In this study, AF was defined as a rapid and irregular atrial rhythm that disrupts normal cardiac pumping function [2]. The 2024 AF management guidelines of the ESC and the European Association of Cardio-Thoracic Surgery (EACTS) were mainly used as the references for selecting patients. The selected patients with atrial fibrillation include paroxysmal atrial fibrillation with a duration of less than or equal to 7 days, as well as persistent atrial fibrillation with a duration of more than 7 days. Patients with left atrial and/or left atrial appendage thrombosis—as determined by transesophageal ultrasound or left atrial CT angiography (CTA)—were excluded before undergoing catheter ablation. Additionally, pulmonary vein CTA was performed to assess the anatomical structure of the patient’s pulmonary veins.

### Exclusion criteria

Patients were excluded from this study based on the following criteria: (1) patients with incomplete clinical data, including missing information on blood tests, clinical biochemistry, or echocardiography; (2) those in whom echocardiography was used to diagnose structural heart diseases such as rheumatic heart disease, congenital heart defects, cardiomyopathy, and valvular heart disease, or those with a history of valve replacement; (3) patients with a history of malignant tumors, acute cardiovascular or cerebrovascular events, acute or chronic infections, or rheumatic immune diseases; (4) patients with secondary AF caused by surgery, hyperthyroidism, myocardial infarction, or other identifiable triggers; (5) patients with a recent history of major surgery, trauma, or blood transfusion; and (6) patients with no follow-up data.

### Radiofrequency catheter ablation with ablation index (RFCA-AI) procedure

The patient was placed in the supine position, and standard aseptic preparation was performed. Local anesthesia was administered via 1% lidocaine infiltration at the femoral vein access site. A puncture was made in the left femoral vein, and a sheath was introduced to facilitate the insertion of mapping electrodes into the coronary sinus. Subsequently, a puncture was made in the right femoral vein, followed by a biatrial septal puncture to advance both sheaths into the left atrium.

A pulmonary vein ring mapping catheter and a cold saline perfusion catheter, integrated with a three-dimensional electroanatomic mapping system, were introduced into the pulmonary veins. Point-by-point ablation was carried out using a power-controlled mode set at 50 W. Linear vestibular ablation of the pulmonary veins was performed under the guidance of the ablation index to achieve complete electrical isolation of the pulmonary veins.

In cases where atrial flutter was detected, additional ablation was performed following detailed mapping, targeting sites such as the tricuspid valve isthmus or the mitral annular region. After a 20-minute post-procedure observation period, electrical conduction between the bilateral pulmonary veins and the left atrium was reassessed to confirm the absence of reconduction. Finally, all catheters were removed, and the puncture site was compressed and bandaged to achieve hemostasis.

### Second-Generation cryoballoon ablation (CBA-2) procedure

The patient was placed in the supine position, and routine aseptic preparation was performed. Local anesthesia was administered via 1% lidocaine infiltration at the femoral vein access site. First, a puncture was made in the left femoral vein, and a sheath was introduced to facilitate the placement of mapping electrodes in the coronary sinus and superior vena cava. Subsequently, the right femoral vein was punctured, and a long sheath along with a J-type guidewire was inserted to facilitate transseptal puncture, allowing access to the left superior pulmonary vein and entry into the left atrium. Time for each freeze was adjusted according to the Time To Isolation (TTI). If TTI < 60 s, PV ostium was frozen for TTI + 120 s, and the time of the second ablation was 120 s.

Bilateral pulmonary venography was performed to assess pulmonary vein anatomy. The cryoballoon delivery sheath was then advanced under the guidance of the J-type guidewire, and a second-generation cryoballoon was introduced. The balloon was sequentially positioned at the left superior, left inferior, right superior, and right inferior pulmonary vein ostia. Following confirmation of adequate occlusion, cryoablation was performed to isolate the pulmonary vein.

During the isolation of the right pulmonary vein, the phrenic nerve was stimulated to monitor diaphragmatic function. In cases where the diaphragmatic movement weakened or ceased, cryoablation was immediately stopped until diaphragmatic movement returned to normal. Upon completion of the procedure, all catheters were removed, and hemostasis was achieved by applying pressure at the puncture site.

### Follow-Up

Postoperative anticoagulation therapy was administered for the first three months, with patients given the option to choose between warfarin or a newer direct oral anticoagulant (DOAC) based on individual preference. The continuation of anticoagulation therapy beyond three months was determined according to the CHA_2_DS_2_-VASc score, assessing the patient’s thromboembolic risk.

Antiarrhythmic medication was discontinued three months post-ablation. Patients were followed up on an outpatient basis at 3, 6, 9, 12, and 15 months postoperatively or earlier if symptoms developed. Each follow-up examination included a 12-lead electrocardiogram (ECG) and 72-hour Holter monitoring to detect arrhythmic events.

In addition, patients were followed up over the telephone once a month to inquire about the recurrence of AF after surgery, assessing clinical symptoms such as palpitations, dyspnea, and fatigue. Patients were advised to undergo an ECG examination promptly in the event of symptom onset.

### Study endpoints

The primary endpoint of the study was the recurrence of AF, defined as the documentation of any rapid trial arrhythmia on a standard 12-lead ECG or an episode lasting > 30 s on the 24-hour Holter monitor, occurring beyond three months post-ablation.

### Statistical analysis

SPSS 25.0 and R 4.2.0 were used for statistical analysis. Categorical variables were expressed as frequency (percentage) and analyzed using the chi-squared test. Normally distributed continuous variables were expressed as mean ± standard deviation (SD), and the comparison of the two groups of data was conducted using the independent sample *t*-test. Data in non-normal distribution were expressed using the median (interquartile range, IQR: 25th–75th percentile) and analyzed using the Mann-Whitney U test.

A receiver operating characteristic (ROC) curve was constructed based on the significant variables identified from univariate analysis results, and the optimal cutoff value was determined using the Youden index to convert continuous variables into binary categorical variables. Multivariate Cox proportional hazards regression analysis was performed to identify independent predictive factors of AF recurrence post-ablation.

A Kaplan-Meier survival curve was generated for the most relevant independent predictor. If the independent risk factor was a categorical variable, the Kaplan-Meier method was used to compare the AF recurrence rate, and the log-rank test was used to determine the statistical significance.

Based on the results of multivariate Cox regression analysis, a nomogram risk prediction model for AF recurrence after catheter ablation was constructed using the column chart method in R 4.2.0. The predictive performance of the model was evaluated using the concordance index (C-index), and its reliability was assessed through calibration curves and decision curve analysis (DCA). Additionally, ROC curves were generated for subgroup analyses to validate the model’s clinical applicability. A *p*-value < 0.05 was considered indicative of statistically significant differences.

## Results

### Baseline population characteristics

A total of 398 patients diagnosed with AF were included in the study. Among them, there were 138 patients (34.7%) with persistent AF, while 270 patients (67.8%) underwent RFCA. Additionally, 143 patients (35.9%) underwent pulmonary vein isolation (PVI), and 81 patients (20.4%) experienced AF recurrence during follow-up.

Comparative analysis between the recurrence group and the non-recurrence group revealed that the duration of AF, recurrence during the blanking period, neutrophil granulocyte count (NE), neutrophil-to-lymphocyte ratio (NLR), red blood cell distribution width (RDW), and left atrial diameter (LAD) were significantly higher among patients in the recurrence group compared to those in the non-recurrence group (*p* < 0.05) (Table 1).

**Table 1:** Clinical baseline characteristics of the study populations with and without recurrences

Clinical Characteristics	No recurrence group (*n* = 317)	Recurrence group (*n* = 81)	*P*-value
Initial value			
Age(years)	62(54–67)	61(54–66)	0.964
Man (%)	196(61.8%)	48(59.3%)	0.672
Height(cm)	167.00(160.00-172.00)	168.00(158.00-173.00)	0.909
Weight(kg)	68.95 ± 11.56	68.24 ± 10.70	0.613
BMI (kg/m^2^)	24.39(22.49–26.89)	24.82(22.97–26.99)	0.723
Smoke (%)	66(20.8%)	21(25.9%)	0.321
Drink (%)	18(5.7%)	7(8.6%)	0.327
PeAF (%)	107(33.8%)	31(38.3%)	0.466
The course of AF (months)	16(5–48)	29(10–84)	0.005
Hypertension (%)	147(46.4%)	34(42.0%)	0.478
Diabetes (%)	45(14.2%)	14(17.3%)	0.485
CHF (%)	7(2.2%)	2(2.5%)	0.888
Stroke/TIA/thromboembolism (%)	7(2.2%)	3(3.7%)	0.443
vascular disease (%)	21(6.6%)	6(7.4%)	0.803
CHD (%)	25(7.9%)	7(8.6%)	0.823
Type of operation			
RFCA (%)	214(67.5%)	56(69.1%)	0.780
Pure pulmonary vein electrical isolation (%)	116(54.2%)	27(48.2%)	0.424
Test			
WBC(*10^9^/L)	5.86(5.04–7.10)	6.02(4.98–7.08)	0.817
NE(*10^9^/L)	3.36(2.78–4.18)	3.96(3.11–4.71)	0.002
LY(*10^9^/L)	1.88(1.60–2.26)	1.91(1.63–2.35)	0.734
NLR	1.73(1.42–2.26)	1.84(1.53–2.51)	0.022
RDW (%)	12.40(12.00–13.00)	12.70(12.20–13.50)	0.019
PLT (10^9^/L)	213.00(180.00-246.00)	207.00(176.50-239.50)	0.629
CRE (umol/L)	75.00(66.00–86.00)	77.00(63.00–87.00)	0.859
UA (umol/L)	370.81 ± 95.06	359.23 ± 88.53	0.322
TC (mmol/L)	4.45 ± 1.02	4.36 ± 1.02	0.472
TG (mmol/L)	1.29(0.97–1.84)	1.28(0.95–1.83)	0.528
HDL (mmol/L)	1.18(1.00-1.46)	1.16(0.97–1.31)	0.279
LDL (mmol/L)	2.92 ± 0.91	2.86 ± 0.99	0.594
NT-proBNP(pg/ml)	196.00(56.50–527.00)	191.00(69.00-540.00)	0.382
DDI (ng/ml)	0.26(0.19–0.37)	0.23(0.20–0.33)	0.273
Examine			
LAD (mm)	36.50(33.30–41.30)	39.60(35.80–44.00)	0.001
LVEDD (mm)	47.38 ± 4.36	48.13 ± 4.16	0.167
LVESD (mm)	29.90(27.65–32.65)	30.10(27.80–32.40)	0.634
EF (%)	65.10(61.80–70.30)	65.80(61.90-70.15)	0.900
CHADS_2_ score			0.911
< 2 n (%)	260(82.0%)	66(81.5%)	
≥ 2 n (%)	57(18.0%)	15(18.5%)	
CHA_2_DS_2_-VASc score			0.988
< 2 n (%)	168(53.0%)	43(53.1%)	
≥ 2 n (%)	149(47.0%)	38(46.9%)	
Blank relapse (%)	12(3.8%)	18(22.2%)	< 0.001

### Determination of the optimal cutoff value using ROC curve analysis

Univariate analysis identified significant factors associated with atrial fibrillation duration, with an optimal cutoff value of 21 months. The corresponding specificity was 52.7%, sensitivity was 65.4%, and the area under the curve (AUC) was 0.601 (95% CI: 0.529–0.672). For NE, the optimal cut-off value was 3.54 *10^9^/L, with a specificity of 59.6%, sensitivity of 63.0%, and an AUC of 0.61 (95%CI = 0.543–0.678). The optimal cutoff value for the NLR was 1.24, with a specificity of 17.7%, sensitivity of 98.9%, and an AUC of 0.582 (95% CI: 0.517–0.648). For RDW, the optimal cutoff value was 12.50%, with a specificity of 38.5%, sensitivity of 72.8%, and an AUC of 0.584 (95% CI: 0.515–0.653). The optimal cutoff value for LAD was 36.75 mm, with a specificity of 52.7%, sensitivity of 70.4%, and an AUC of 0.624 (95% CI: 0.557–0.692).

**Fig. 1:**
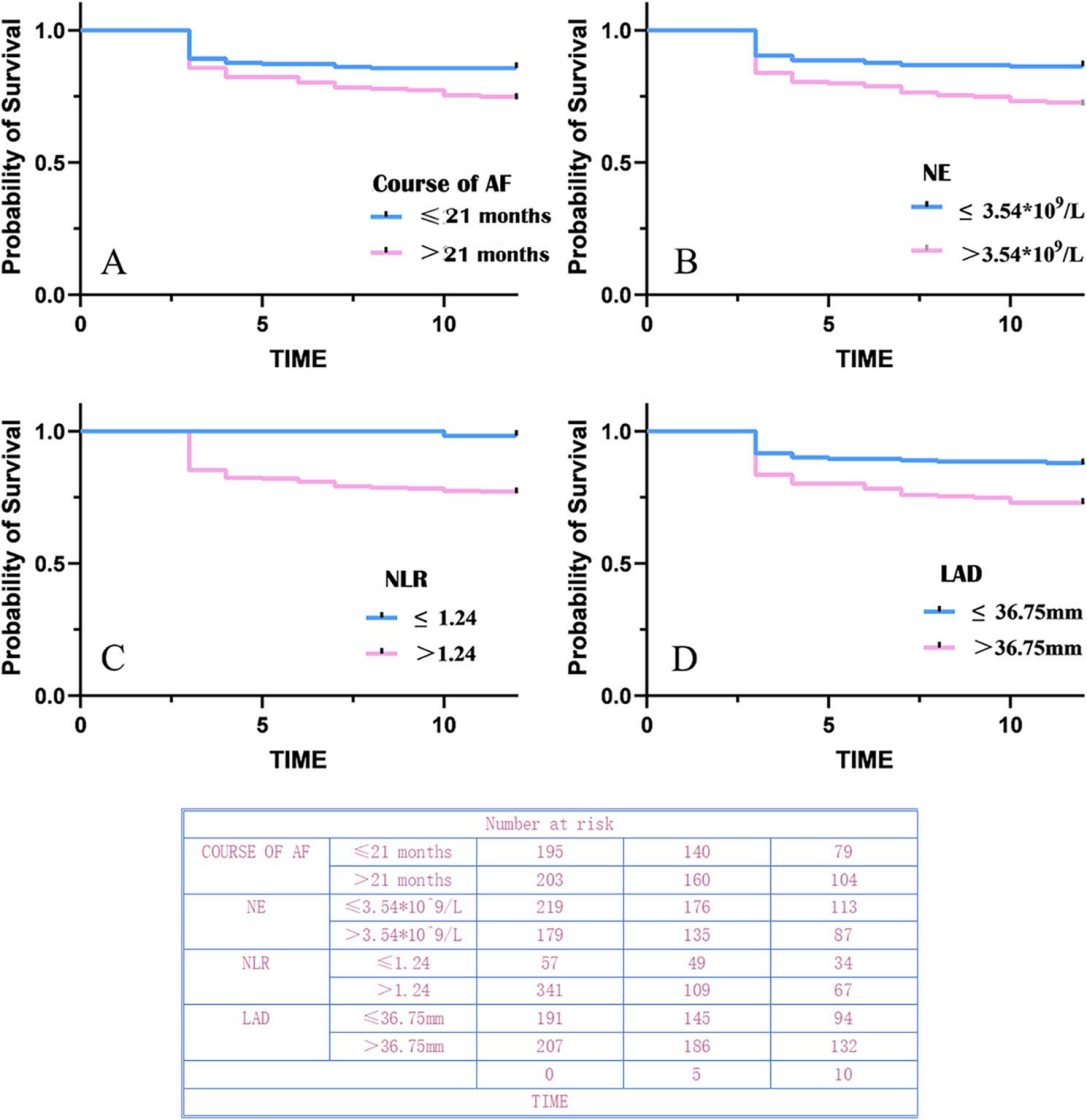
Differences in clinical parameters between the recurrence and non-recurrence groups. (**A**) Duration of atrial fibrillation. (**B**) Neutrophil count. (**C**) Neutrophil-to-lymphocyte ratio. (**D**) Red blood cell distribution width. (**E**) Left anterior and posterior atrial diameter The areas under the ROC curves (AUC) for all five factors were > 0.5, and all *p*-values were < 0.05, indicating statistically significant differences

All five variables—atrial fibrillation duration, NE, NLR, RDW, and LAD—demonstrated statistical significance (*p* < 0.05). The AUC values for these variables exceeded 0.5, and all *p*-values were < 0.05, indicating statistically significant differences (Table 2; Fig. 1).

**Table 2:** Significant differences in ROC curves between the recurrence and non-recurrence groups

Clinical Characteristics	AUC	*P*-value	95.0% CI	Optimum cutoff value
Course of AF	0.601	0.005	0.529–0.672	21
NE	0.610	0.002	0.543–0.678	3.54
NLR	0.582	0.022	0.517–0.648	1.24
RDW	0.584	0.020	0.515–0.653	12.50
LAD	0.624	0.001	0.557–0.692	36.75

### Multivariate analysis

AF duration, NE, NLR, RDW, and LAD were converted into binary variables based on their respective optimal cutoff values. Multivariate Cox proportional hazards regression analysis identified the duration of AF, recurrence during the blanking period, NE, NLR, and LAD as independent risk factors for AF recurrence (*p* < 0.05). However, RDW did not demonstrate statistical significance (*p* > 0.05) (Table 3). Considering the partial overlap between NE and NLR, a multicollinearity analysis was conducted in this study, and it was concluded that the VIF of NE was 10,814 and that of NLR was 1.811. Meanwhile, multicollinearity analysis was conducted on all the predictors obtained from the multi-factor analysis, and there were no serious collinear problems.

**Table 3:** Multivariate COX regression analysis of recurrence after catheter ablation

Clinical Characteristics	B	SE	Wald	*p* value	HR	95.0% CI
Course of AF	0.626	0.234	7.184	0.007	1.873	1.183–2.957
NE	0.510	0.250	4.153	0.042	1.665	1.020–2.718
NLR	2.365	1.016	5.419	0.020	10.642	1.453–77.942
RDW	0.477	0.273	3.065	0.080	1.611	0.945–2.749
LAD	0.983	0.264	13.915	< 0.001	2.673	1.595–4.482
Blank relapse	1.474	0.269	29.984	< 0.001	4.369	2.577–7.406

### Survival analysis of independent risk factors for postoperative AF recurrence

Based on the results of the Cox regression results, RDW was excluded. The remaining independent risk factors (AF duration, NE, NLR, and LAD) were stratified according to their optimal cutoff values and analyzed using the Kaplan-Meier (KM) method with the log-rank test. The results demonstrated statistically significant differences in AF recurrence among different stratifications (*p* < 0.05).

**Fig. 2:**
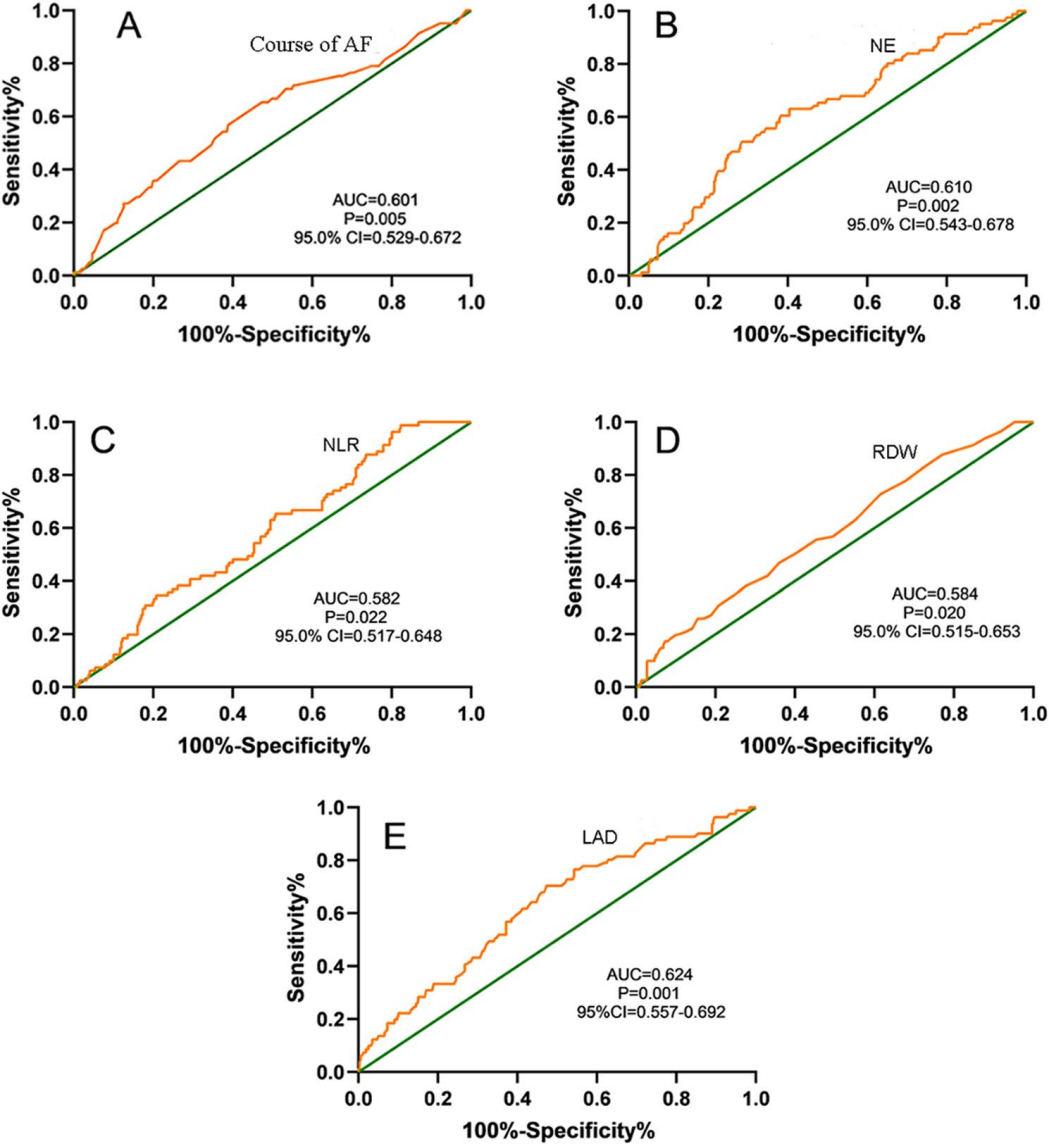
Kaplan-Meier (KM) survival curves of independent risk factors for postoperative AF recurrence. (**A**) AF duration; (**B**) NE; (**C**) NLR; (**D**) LAD The effects of AF duration, NE, NLR, and LAD on post-ablation AF recurrence were statistically significant across different stratifications. As per the KM curves, the risk of post-ablation AF recurrence increased with longer AF duration. Additionally, patients with NE > 3.54 × 10^9^/L, NLR > 1.24, and LAD > 36.75 mm exhibited a significantly higher risk of post-ablation AF recurrence

The KM survival curves for the four independent risk factors indicated that a longer AF duration was associated with a higher risk of AF recurrence following ablation. Furthermore, patients with NE > 3.54 × 10^9^/L, NLR > 1.24, and LAD > 36.75 mm exhibited an increased risk of AF recurrence post-ablation (Table 4; Fig. 2).

**Table 4:** Survival analysis of recurrence after catheter ablation

Clinical Characteristics	Median relapse time	Standard error	95% confidence interval		*P*-value
			lower limit	upper limit	
Course of AF					0.004
≤ 21	10.800	0.213	10.382	11.218	
> 21	10.143	0.243	9.667	10.619	
NE					<0.001
≤ 3.54	10.881	0.194	10.501	11.262	
> 3.54	9.955	0.268	9.430	10.481	
NLR					<0.001
≤ 1.24	11.965	0.035	11.897	12.033	
> 1.24	10.214	0.187	9.848	10.580	
LAD					<0.001
≤ 36.75	11.031	0.200	10.639	11.424	
> 36.75	9.942	0.250	9.452	10.432	

### The nomogram prediction model

**Fig. 3:**
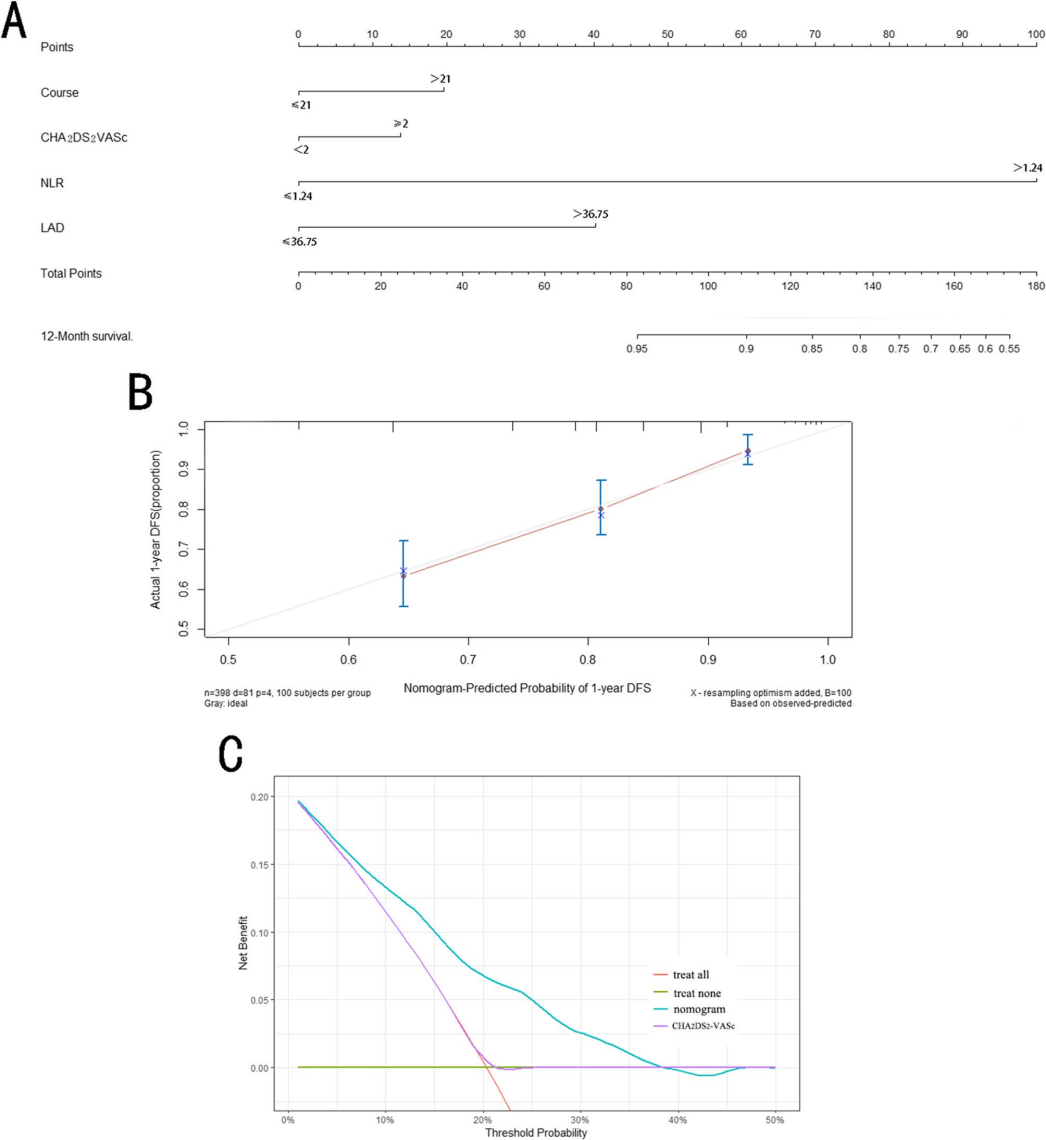
Nomogram prediction model and validation analyses. (** A**) The nomogram prediction model for** AF **recurrence post-ablation. AF duration represents the duration of** AF**, with a cutoff of 21 months, assigned 20 points in the nomogram. NLR represents the ratio of neutrophils to lymphocytes and is dichotomized at 1.24, assigned 100 points. The LAD truncation value is 36.75 mm and is assigned 40 points. The CHA_2_DS_2_-VASc score is categorized as ≥ 2 points and assigned 15 points. The total score is derived from the sum of these values, and the 12-month post-ablation recurrence rate is predicted accordingly. According to the results, as** AF **duration, NLR and LAD increase, and the CHA_2_DS_2_-VASc score is ≥ 2 points, the total score increases, correlating with a higher AF recurrence rate post-ablation.(** B **) Calibration curve of the nomogram prediction model. The consistency index (C-index) of the model is 0.707 (95% CI: 0.566–0.847), indicating moderate predictive accuracy. The calibration curve demonstrates good agreement between the predicted and actual probabilities of AF recurrence, supporting the model’s reliability. (**C**) DCA comparing the nomogram with the CHA_2_DS_2_-VASc score. The DCA curve of the nomogram consistently lies above that of the CHA_2_DS_2_-VAS score, indicating that the nomogram model has better clinical utility and enhanced predictive performance

Based on the multivariate Cox proportional hazards regression analysis, a nomogram prediction model was developed incorporating the duration of AF, NLR, LAD, and CHA_2_DS_2_-VASc score. The calibration curve demonstrated strong concordance between the predicted and observed recurrence rates. Additionally, the predictive accuracy of the nomogram was significantly higher than that of the CHA_2_DS_2_-VASc score. The results indicate that the nomogram model exhibits robust clinical applicability, outperforming the CHA_2_DS_2_-VASc score in predicting AF recurrence (Table 5; Fig. 3). In this study, the model credibility was evaluated by the Bootstrap method (B = 500), and the results showed that the model had good stability. At the same time, it has moderate prediction accuracy (C Index = 0.65).

**Table 5:** Nomogram c-index

Model	C-index(95%CI)
CHA_2_DS_2_-VASc	0.499(0.359–0.640)
Course + CHA_2_DS_2_-VASc + NLR + LAD	0.707(0.566–0.847)

### Subgroup analysis

**Fig. 4:**
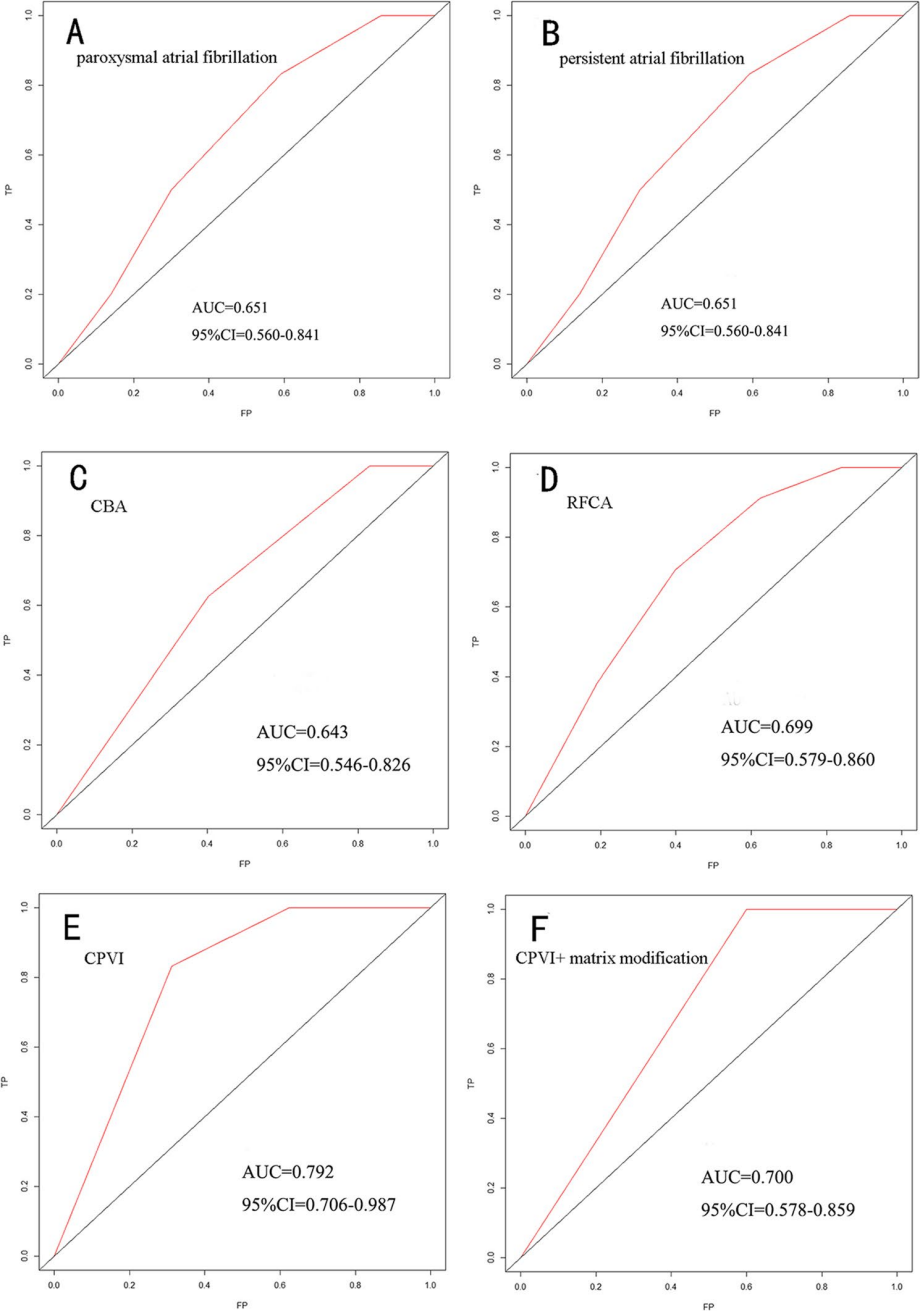
ROC curve analysis for AF subtypes and surgical methods. (**A**) Paroxysmal AF; (**B**) Persistent AF. The AUC for these two subgroups is 0.651 (95% CI: 0.560–0.841), indicating that the nomogram is applicable for both subtypes. ROC curve analysis for different surgical methods:(** C**). CBA;(** D**) RFCA;(** E**) CPVI;(** F**) CPVI + matrix modification. The AUC for the RFCA group is 0.699 (95% CI: 0.579–0.860) and 0.643 (95% CI: 0.546–0.826) for the CBA group; the AUC for the CPVI group is 0.792 (95% CI: 0.706–0.987), and 0.700 (95% CI: 0.578–0.859) for the CPVI + matrix modification group. These findings indicate that the nomogram prediction model is applicable across different AF subtypes and ablation strategies

To assess the applicability of the nomogram model across different patient subgroups, the study population was stratified based on AF type and ablation technique:

AF Type Subgroup Analysis: Patients were categorized into paroxysmal AF and persistent AF groups, and the respective ROC curves were constructed. The results confirmed that the nomogram model remained applicable across different AF subtypes (Fig. 4).

Ablation Procedure Subgroup Analysis: Patients were classified into radiofrequency ablation (RFA) and cryoballoon ablation (CBA) groups. Notably, patients with persistent AF underwent RFA. Further stratification of the RFA group was performed based on the ablation strategy, and patients were divided into those undergoing PVI alone and those receiving PVI combined with substrate modification. ROC curve analysis confirmed that the nomogram model was applicable across different ablation techniques (Fig. 4).

## Discussion

This study found that the duration of atrial fibrillation, recurrence during the ablation period, NE, NLR and LAD were identified as independent risk factors for the recurrence of atrial fibrillation after catheter ablation.

In 2014, Letsas et al. [8] conducted postoperative follow-up of 126 patients with paroxysmal AF and found that both CHADS_2_ and CHA_2_DS_2_-VASc scores ≥ 2 had the highest predictive value for recurrence of AF. These scores were identified as independent predictors of postoperative recurrence after AF ablation. This study highlighted the predictive value of CHADS_2_ and CHA_2_DS_2_-VaSc scores in assessing the risk of AF recurrence post-ablation. However, it is important to note that this study had several limitations: it specifically focused on patients with paroxysmal AF, involved a small sample size, and incorporated heterogenous antiarrhythmic therapies. In several studies in China and other countries, it has been concluded that CHADS_2_ and CHA_2_DS_2_-VASc scores have predictive value for postoperative AF recurrence of post-ablation [9]. However, the results of our study suggest that these scores do not have statistical significance in predicting AF recurrence after ablation, as evidenced by *p-*values > 0.05.

The potential association between inflammation and AF was first identified in a study conducted in 1997 [10]. Since then, the relationship between inflammation and AF has been explored in several studies [11]. In 2018, Zhang et al. [12] proposed a new evaluation system for predicting the postoperative recurrence probability of AF based on an inflammatory mechanism. This system integrated multiple inflammatory factors, such as albumin, neutrophils, and platelets, to assess the risk of AF recurrence. Neutrophils and lymphocytes are two important subtypes among the components of leukocyte composition.

In recent years, the NLR has been recognized as a new biomarker for inflammation, combining two distinct immune pathways: a long-term immune response mediated by lymphocytes and a rapid immune response reflected by neutrophils. NLR has been shown to be positively correlated with inflammatory responses, providing a more comprehensive reflection of the degree and integrity of systemic inflammation. Compared to individual inflammatory markers, NLR offers a more reliable and stable assessment of inflammation, as it is less susceptible to conventional physiological conditions [13]. 

Acet et al. retrospectively analyzed data and demonstrated that the NLR value was significantly higher in the AF group compared to the non-AF group [14]. Similarly, Shao et al. concluded that NLR is significantly associated with high-sensitivity C-reactive protein (hs-CRP), suggesting that NLR may serve as a risk factor for AF [15]. In a retrospective study conducted in 2015, it was further found that NLR could be a predictor for the recurrence of AF after medical cardioversion [16]. Our study results also support these findings, suggesting that NLR is an important predictor of AF recurrence following ablation. Specifically, NLR > 1.24 was found to be associated with a higher risk of postoperative AF recurrence, with a higher risk of recurrence correlating with an increasing NLR value. Notably, the nomogram model developed in this study highlighted the high predictive value of NLR, which has the highest value, indicating that it had the largest proportion in predicting AF recurrence, further establishing its utility as a critical predictive tool.

Among the mechanisms underlying the occurrence and maintenance of AF, structural remodeling has garnered considerable attention from researchers. Left atrial enlargement represents one of the important manifestations of atrial structural remodeling and results from the combined effects of various pathological factors on the left atrium. In a Canadian study, a left atrial internal diameter >45 mm was identified as one of the potential causes of AF progression, suggesting that left atrial enlargement is an important risk factor in patients with AF [17]. Furthermore, results from a study conducted in 2019 demonstrated that left atrial enlargement serves as an independent predictor of AF recurrence after surgery [18]. Recent studies have found that atrial fibrosis and structural remodeling (such as extracellular matrix deposition and interstitial fibrosis) also play a key role in the initiation and maintenance of AF. Fibrosis provides an anatomical basis for reentry by increasing atrial conduction heterogeneity and forming electrical conduction block areas. Ion channel dysfunction and fibrosis do not exist in isolation but promote each other through common pathological mechanisms such as oxidative stress and inflammatory responses. In the present study, we found a direct correlation between an increase in the LAD and a higher recurrence rate of AF post-ablation, suggesting that left atrial size is a reliable predictor of AF recurrence following ablation.

In addition, the duration of AF plays a significant role in the recurrence rate of AF. As the duration of AF increases, the likelihood of AF recurrence also rises [19]. The underlying mechanism may be attributed to the increased probability of both atrial electrical remodeling and structural remodeling over time, which becomes progressively more irreversible. Thus, the AF recurrence rate was significantly higher in patients with a prolonged duration of the arrhythmia. In our study, AF duration was identified as one of the independent risk factors for AF recurrence post-ablation.

In this study, statistical analyses of various clinical variables of patients helped to determine that the duration of AF, recurrence during the blanking period, NE, NLR, and LAD were independent risk factors for AF recurrence post-ablation. The purpose of the prediction model developed in this study was to estimate the postoperative recurrence probability of AF prior to the operation. Since recurrence during the blanking period serves as a postoperative index, it was excluded from the prediction model.

While NE serves as a useful marker of inflammation, NLR provides a more comprehensive view of the inflammatory process as it reflects both a prolonged immune response dominated by lymphocytes and a rapid immune response mediated by neutrophils. Additionally, NLR is less susceptible to detection interference, making it a more reliable indicator. Therefore, a nomogram prediction model was constructed integrating the CHA_2_DS_2_-VASc score and the three independent risk factors, namely, AF duration, NLR, and LAD.

Multiple statistical verification results demonstrated that this nomogram prediction model has significant predictive value and clinical applicability for AF recurrence post-ablation, outperforming the CHA_2_DS_2_-VASc score in terms of prediction accuracy. Previous studies have shown that the value of traditional scores in predicting prognosis is limited. The prediction model constructed in this study, although the C-index indicates moderate prediction accuracy, has the advantages of richer content and a wider range of predictive factors compared with traditional scores. The predicted C-index is also greater than that of traditional scores and has high clinical value. Furthermore, the model was found to be applicable to various types of AF and different ablation procedures. By providing an individualized assessment of the risk of AF recurrence post-ablation, the nomogram prediction model can help in optimizing treatment plans, reducing treatment costs, and enabling each patient to select the most suitable treatment approach. This study used a single-center cohort with a small sample size, which limited its external validity. Future studies can further confirm its external validity by incorporating broader population characteristics and external cohorts.

## Limitations

This study has several limitations. First, as a single-center study with a relatively small sample size, its generalizability is limited. A multicenter, large-sample study is required for further validation. Due to the small sample size, the CHA2DS2-VASc score was analyzed in grouped categories rather than as an absolute continuous variable, which may have influenced the predictive power of the model. Secondly, the incidence of AF recurrence following ablation may have been underestimated due to the potential presence of asymptomatic recurrences, which might not have been fully captured despite the study’s requirement that patients participate in a postoperative 72-hour Holter monitoring. Future studies should consider the use of more accurate or extended monitoring strategies to assess patients’ postoperative heart rhythm. Future studies should consider the use of more accurate or extended monitoring strategies to assess patients’ postoperative heart rhythm. Lastly, since the exclusion criteria include a history of malignant tumors, secondary AF, recent major surgeries, etc., this may introduce selection bias and may affect the universality of the results.

## Conclusion

The duration of AF, recurrence during the blanking period, NE, NLR, and LAD were identified as independent risk factors for AF recurrence following catheter ablation. Based on these findings, a nomogram prediction model was developed, integrating the CHA_2_DS_2_-VASc score with AF duration, NLR, and LAD, to predict the 12-month recurrence probability post-ablation. Among these factors, NLR had the highest predictive value, followed by LAD, duration of AF, and CHA_2_DS_2_-VASc score. The nomogram-derived total score increased with prolonged AF duration, elevated NLR, higher LAD, and CHA_2_DS_2_-VASc scores ≥ 2, correlating with a higher risk of AF recurrence post-ablation. This nomogram prediction model demonstrated greater clinical predictive accuracy and applicability compared to the CHA_2_DS_2_-VASc score alone. Additionally, it exhibited robust clinical predictive value for AF recurrence across AF subtypes (persistent and paroxysmal AF) and various ablation procedures (radiofrequency ablation, cryoablation, pulmonary vein isolation, and pulmonary vein isolation with matrix modification).

## Data Availability

The datasets used or analysed during the current study are available from the corresponding author on reasonable request.

## Abbreviations

AF: Atrial Fibrillation
BMI: Body Mass Index
CBA: Cryo-balloon Ablation
RFCA: Radiofrequency Ablation
CPVI: Circumferential Pulmonary Vein Isolation
CTA: Computerized Tomography Angiography
ESC: European Society of Cardiology
NE: Neutrophilicgranulocyte
NLR: Ratio of neutrophils to lymphocytes
RDW: Red blood cell distribution width
CRP: C-reactive protein
NT-proBNP: N-terminal pro-B-type Natriuretic Peptide
MHR: Monocyte/HDL-C Ratio
LAD: Left Atrial Diameter
LVEDD: Left Ventricular End-diastolic Diameter
LVESD: Left Ventricular End-systolic Diameter
LVEF: Left Ventricular Ejection Fraction
RFCA-AI: RadiofrequencyAblation-AblationIndex
CBA-2: Second-generationCryo-balloonAblation
ECG: electrocardiogram
